# [Bis(3-amino­prop­yl)amine-κ^3^ *N*,*N*′,*N*′′]bis­(thio­cyanato-κ*N*)cobalt(II)

**DOI:** 10.1107/S1600536811025876

**Published:** 2011-07-06

**Authors:** Jan Boeckmann, Christian Näther

**Affiliations:** a Institut für Anorganische Chemie, Christian-Albrechts-Universität Kiel, Max-Eyth Strasse 2, D-24098 Kiel, Germany

## Abstract

The asymmetric unit of the title compound, [Co(NCS)_2_(C_6_H_17_N_3_)], consists of one Co^2+^ cation, two thio­cyanate anions and one bis­(3-amino­prop­yl)amine ligand, all in general positions. The cobalt cation is coordinated by five N atoms of two terminal N-bonded thio­cyanate anions and one bis­(3-amino­prop­yl)amine ligand, defining a slightly distorted square-pyramidal coordination polyhedron. The mol­ecules are held together in the crystal by weak N—H⋯S inter­actions.

## Related literature

For isostructural compounds with copper(II) and cadmium(II) but with an alternate setting of the space group, see: Cannas *et al.* (1974 ▶, 1977 ▶). For background to thermal decomposition reactions and the resulting inter­mediates, see: Boeckmann & Näther (2010 ▶, 2011 ▶); Boeckmann *et al.* (2011 ▶); Wöhlert *et al.* (2011 ▶); Wriedt *et al.* (2009*a* ▶,*b* ▶); Wriedt & Näther (2010 ▶).
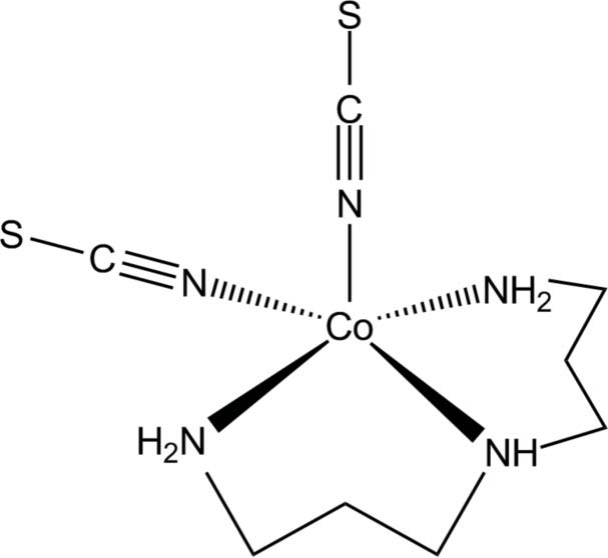


## Experimental

### 

#### Crystal data

[Co(NCS)_2_(C_6_H_17_N_3_)]
*M* *_r_* = 306.32
Monoclinic, 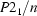

*a* = 7.5515 (4) Å
*b* = 14.2250 (11) Å
*c* = 12.8825 (8) Å
β = 103.091 (7)°
*V* = 1347.88 (15) Å^3^
*Z* = 4
Mo *K*α radiation
μ = 1.57 mm^−1^
*T* = 170 K
0.11 × 0.08 × 0.06 mm

#### Data collection

Stoe IPDS-1 diffractometer
Absorption correction: numerical (*X-SHAPE* and *X-RED32*; Stoe & Cie, 2008 ▶) *T*_min_ = 0.856, *T*_max_ = 0.905
15883 measured reflections
3234 independent reflections
2832 reflections with *I* > 2σ(*I*)
*R*_int_ = 0.043

#### Refinement

*R*[*F* ^2^ > 2σ(*F* ^2^)] = 0.027
*wR*(*F* ^2^) = 0.070
*S* = 1.04
3234 reflections
146 parameters
H-atom parameters constrained
Δρ_max_ = 0.33 e Å^−3^
Δρ_min_ = −0.51 e Å^−3^

### 

Data collection: *X-AREA* (Stoe & Cie, 2008 ▶); cell refinement: *X-AREA*; data reduction: *X-AREA*; program(s) used to solve structure: *SHELXS97* (Sheldrick, 2008 ▶); program(s) used to refine structure: *SHELXL97* (Sheldrick, 2008 ▶); molecular graphics: *XP* in *SHELXTL* (Sheldrick, 2008 ▶) and *DIAMOND* (Brandenburg, 2011 ▶); software used to prepare material for publication: *SHELXL97*.

## Supplementary Material

Crystal structure: contains datablock(s) I, global. DOI: 10.1107/S1600536811025876/bt5568sup1.cif

Structure factors: contains datablock(s) I. DOI: 10.1107/S1600536811025876/bt5568Isup2.hkl

Additional supplementary materials:  crystallographic information; 3D view; checkCIF report

## Figures and Tables

**Table 1:** Hydrogen-bond geometry (Å, °)

*D*—H⋯*A*	*D*—H	H⋯*A*	*D*⋯*A*	*D*—H⋯*A*
N1—H1*A*⋯S21^i^	0.92	2.82	3.6177 (14)	145
N1—H1*B*⋯S11^ii^	0.92	2.80	3.5665 (14)	142
N2—H2⋯S11^iii^	0.93	2.69	3.5903 (15)	162
N3—H3*A*⋯S21^iv^	0.92	2.69	3.5839 (16)	165

Symmetry codes: (i) 
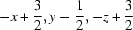
; (ii) 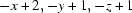; (iii) 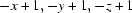; (iv) 
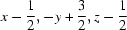
.

**Fig. 1.**
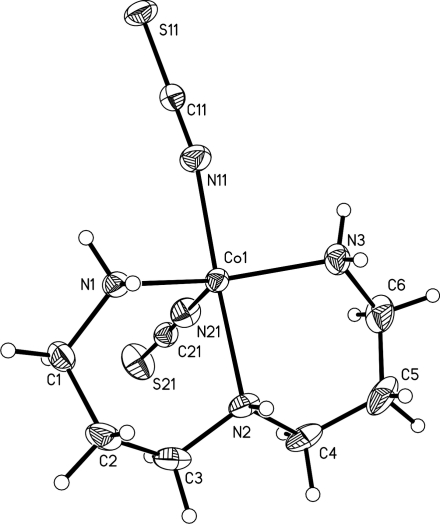
: Crystal structure of the title compound, showing the coordination around the Co2+ cations with labelling and displacement ellipsoids drawn at the 50% probability level.

**Fig. 2.**
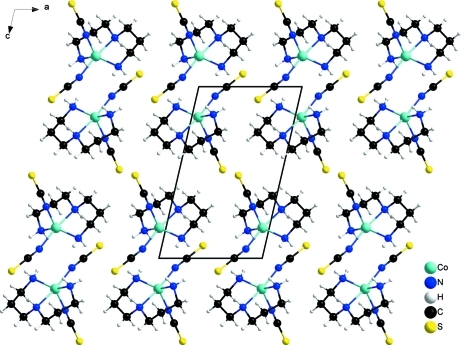
: Packing diagram of the title compound with view along the crystallographic b axis (aqua = cobalt; yellow = sulfur; blue = nitrogen; black = carbon; light-grey = hydrogen).

## supplementary crystallographic information

### Comment

Recently, we reported about thermal decomposition reactions as an alternative synthetic strategy for the rational design of condensed frameworks (Wriedt *et al.* (2009*a*,*b*); Wriedt & Näther (2010)). Herein, transition metal(II) thio- and selenocyanato coordination compounds with different neutral *N*-donor co-ligands are heated, which leads to a stepwise loss of the neutral co-ligands and to the formation of ligand-deficient intermediates. Depending on the precursor, the dimensionality of the resulting intermediates can simply be predefined. If precursors are used, which consist of bidentate co-ligands, two-dimensional structures can be obatined, in which the metal(II) cations are octahedrally coordinated by four bridging anions and one bridging co-ligand (Wöhlert *et al.* (2011); Wriedt *et al.* (2009*a*,*b*); Wriedt & Näther (2010)). If precursor complexes with monodentate co-ligands are used, one-dimensional structures are obtained in which the metal(II) cations are only bridged *via* the anionic ligands (Boeckmann & Näther (2010); Boeckmann & Näther (2011); Boeckmann *et al.* (2011)). In further work we tried to synthesize octahedral coordinated precursor compounds based on the tridentate co-ligand bis(3-aminopropyl)amin in combination with a volatile monodentate co-ligand like e.g. water or methanol, which on heating should transform into dimers. Surprisingly a five-coordinated complex was obtained which was characterized by single crystal X-ray diffraction.

In the crystal structure the cobalt(II) cations are coordinated by five nitrogen atoms of two terminal *N*-bonded thiocyanate anions and one tridenate co-ligand bis(3-aminopropyl)amin in a slightly distorted square-pyramidal coordination geometry (Fig. 1). The title compound is isostrucutral to its copper(II) and cadmium(II) thiocyanato compounds reported recently (Cannas *et al.* (1974); Cannas *et al.* (1977)). These discrete complexes are arranged into columns which elongated in the direction of the crystallographic *a* axis (Fig. 2). Each two of these columns are pairwise centrosymmetrically arranged into a three-dimensional packing. The molecules are held together by weak N-H···S interactions.

### Experimental

The title compound was prepared by the reaction of 96.6 mg Co(NCS)_2_^.^H_2_O (0.50 mmol), 40.4 µ*L* pyridine (0.50 mmol) and 70.6 µ*L* bis(3-aminopropyl)amine (0.50 mmol) in 1.50 ml water at RT in a closed 3 ml snap cap vial. After one week violet blocks of the title compound were obtained.

### Refinement

All H atoms were located in difference map but were positioned with idealized geometry and were refined isotropically with *U*_eq_(H) = 1.2 *U*_eq_(C) and *U*_eq_(H) = 1.2 *U*_eq_(N) of the parent atom using a riding model with C—H = 0.99 Å, N—H = 0.93 Å (NH_1_) and N—H = 0.92 Å (NH_2_).

### Crystal data

**Table:**

[Co(NCS)_2_(C_6_H_17_N_3_)]	*F*(000) = 636
*M**_r_* = 306.32	*D*_x_ = 1.509 Mg m^−^^3^
Monoclinic, *P*2_1_/*n*	Mo *K*α radiation, λ = 0.71073 Å
Hall symbol: -P 2yn	Cell parameters from 8000 reflections
*a* = 7.5515 (4) Å	θ = 3.2–28.1°
*b* = 14.2250 (11) Å	µ = 1.57 mm^−^^1^
*c* = 12.8825 (8) Å	*T* = 170 K
β = 103.091 (7)°	Block, violet
*V* = 1347.88 (15) Å^3^	0.11 × 0.08 × 0.06 mm
*Z* = 4	

### Data collection

**Table:**

Stoe IPDS-1 diffractometer	3234 independent reflections
Radiation source: fine-focus sealed tube	2832 reflections with *I* > 2σ(*I*)
graphite	*R*_int_ = 0.043
φ scans	θ_max_ = 28.1°, θ_min_ = 3.2°
Absorption correction: numerical (*X-SHAPE* and *X-RED32*; Stoe & Cie, 2008)	*h* = −9→9
*T*_min_ = 0.856, *T*_max_ = 0.905	*k* = −18→18
15883 measured reflections	*l* = −16→17

### Refinement

**Table:**

Refinement on *F*^2^	Secondary atom site location: difference Fourier map
Least-squares matrix: full	Hydrogen site location: inferred from neighbouring sites
*R*[*F*^2^ > 2σ(*F*^2^)] = 0.027	H-atom parameters constrained
*wR*(*F*^2^) = 0.070	*w* = 1/[σ^2^(*F*_o_^2^) + (0.0414*P*)^2^ + 0.3919*P*] where *P* = (*F*_o_^2^ + 2*F*_c_^2^)/3
*S* = 1.04	(Δ/σ)_max_ = 0.002
3234 reflections	Δρ_max_ = 0.33 e Å^−^^3^
146 parameters	Δρ_min_ = −0.51 e Å^−^^3^
0 restraints	Extinction correction: *SHELXL97* (Sheldrick, 2008), Fc^*^=kFc[1+0.001xFc^2^λ^3^/sin(2θ)]^-1/4^
Primary atom site location: structure-invariant direct methods	Extinction coefficient: 0.0069 (15)

### Special details

**Table:**

Geometry. All esds (except the esd in the dihedral angle between two l.s. planes) are estimated using the full covariance matrix. The cell esds are taken into account individually in the estimation of esds in distances, angles and torsion angles; correlations between esds in cell parameters are only used when they are defined by crystal symmetry. An approximate (isotropic) treatment of cell esds is used for estimating esds involving l.s. planes.
Refinement. Refinement of F^2^ against ALL reflections. The weighted R-factor wR and goodness of fit S are based on F^2^, conventional R-factors R are based on F, with F set to zero for negative F^2^. The threshold expression of F^2^ > 2sigma(F^2^) is used only for calculating R-factors(gt) etc. and is not relevant to the choice of reflections for refinement. R-factors based on F^2^ are statistically about twice as large as those based on F, and R- factors based on ALL data will be even larger.

### Fractional atomic coordinates and isotropic or equivalent isotropic displacement parameters (Å^2^)

**Table:**

	*x*	*y*	*z*	*U*_iso_*/*U*_eq_	
Co1	0.56529 (3)	0.529621 (13)	0.677913 (16)	0.01799 (9)	
N1	0.70311 (18)	0.40562 (9)	0.66179 (10)	0.0210 (3)	
H1A	0.6229	0.3651	0.6195	0.025*	
H1B	0.7930	0.4192	0.6264	0.025*	
N2	0.42397 (18)	0.45541 (10)	0.78508 (11)	0.0231 (3)	
H2	0.3396	0.4158	0.7426	0.028*	
N3	0.32593 (19)	0.60251 (10)	0.61765 (11)	0.0260 (3)	
H3A	0.3495	0.6483	0.5722	0.031*	
H3B	0.2434	0.5615	0.5779	0.031*	
C1	0.7876 (2)	0.35590 (11)	0.76221 (14)	0.0265 (3)	
H1C	0.8689	0.3995	0.8108	0.032*	
H1D	0.8619	0.3028	0.7463	0.032*	
C2	0.6429 (3)	0.31937 (12)	0.81595 (14)	0.0300 (4)	
H2A	0.7001	0.2748	0.8727	0.036*	
H2B	0.5521	0.2839	0.7629	0.036*	
C3	0.5447 (3)	0.39473 (13)	0.86483 (13)	0.0301 (4)	
H3C	0.4711	0.3640	0.9097	0.036*	
H3D	0.6362	0.4348	0.9119	0.036*	
C4	0.3199 (3)	0.51944 (14)	0.84044 (15)	0.0349 (4)	
H4A	0.4057	0.5638	0.8851	0.042*	
H4B	0.2627	0.4817	0.8886	0.042*	
C5	0.1732 (3)	0.57537 (16)	0.76589 (18)	0.0420 (5)	
H5A	0.0926	0.5308	0.7182	0.050*	
H5B	0.0987	0.6078	0.8090	0.050*	
C6	0.2402 (3)	0.64767 (13)	0.69795 (16)	0.0355 (4)	
H6A	0.1368	0.6870	0.6611	0.043*	
H6B	0.3298	0.6892	0.7442	0.043*	
N11	0.6789 (2)	0.58506 (10)	0.55469 (12)	0.0293 (3)	
C11	0.7699 (2)	0.61541 (10)	0.50112 (12)	0.0199 (3)	
S11	0.89760 (6)	0.65832 (3)	0.42551 (3)	0.03094 (12)	
N21	0.70829 (19)	0.60345 (10)	0.80127 (12)	0.0274 (3)	
C21	0.7925 (2)	0.63897 (11)	0.87783 (13)	0.0225 (3)	
S21	0.91711 (7)	0.68852 (4)	0.98333 (4)	0.03999 (14)	

### Atomic displacement parameters (Å^2^)

**Table:**

	*U*^11^	*U*^22^	*U*^33^	*U*^12^	*U*^13^	*U*^23^
Co1	0.01799 (12)	0.01756 (12)	0.01939 (12)	0.00138 (7)	0.00625 (8)	0.00026 (7)
N1	0.0237 (6)	0.0202 (6)	0.0208 (6)	0.0026 (5)	0.0087 (5)	0.0013 (5)
N2	0.0225 (6)	0.0295 (7)	0.0195 (6)	−0.0052 (5)	0.0092 (5)	−0.0009 (5)
N3	0.0243 (7)	0.0301 (7)	0.0233 (7)	0.0076 (5)	0.0046 (5)	−0.0032 (5)
C1	0.0242 (8)	0.0232 (7)	0.0312 (9)	0.0032 (6)	0.0045 (6)	0.0086 (6)
C2	0.0363 (9)	0.0226 (8)	0.0311 (9)	−0.0039 (6)	0.0074 (7)	0.0103 (6)
C3	0.0369 (9)	0.0345 (9)	0.0202 (8)	−0.0070 (7)	0.0096 (7)	0.0065 (6)
C4	0.0370 (10)	0.0456 (10)	0.0297 (9)	−0.0021 (8)	0.0235 (8)	−0.0041 (7)
C5	0.0280 (9)	0.0571 (13)	0.0476 (12)	0.0059 (8)	0.0227 (8)	−0.0088 (9)
C6	0.0339 (9)	0.0371 (9)	0.0370 (10)	0.0144 (7)	0.0111 (8)	−0.0093 (8)
N11	0.0329 (8)	0.0246 (7)	0.0344 (8)	0.0023 (5)	0.0163 (6)	0.0078 (6)
C11	0.0211 (7)	0.0172 (6)	0.0210 (7)	0.0004 (5)	0.0039 (6)	−0.0016 (5)
S11	0.0279 (2)	0.0411 (2)	0.0277 (2)	−0.00521 (17)	0.01443 (17)	0.00238 (17)
N21	0.0255 (7)	0.0272 (7)	0.0292 (7)	−0.0064 (5)	0.0053 (6)	−0.0037 (6)
C21	0.0211 (7)	0.0203 (7)	0.0275 (8)	−0.0004 (5)	0.0081 (6)	0.0015 (6)
S21	0.0455 (3)	0.0373 (3)	0.0297 (2)	0.0007 (2)	−0.0071 (2)	−0.00828 (18)

### Geometric parameters (Å, °)

**Table:**

Co1—N21	2.0043 (14)	C2—C3	1.519 (3)
Co1—N3	2.0758 (13)	C2—H2A	0.9900
Co1—N1	2.0822 (13)	C2—H2B	0.9900
Co1—N11	2.1201 (14)	C3—H3C	0.9900
Co1—N2	2.1970 (13)	C3—H3D	0.9900
N1—C1	1.486 (2)	C4—C5	1.517 (3)
N1—H1A	0.9200	C4—H4A	0.9900
N1—H1B	0.9200	C4—H4B	0.9900
N2—C3	1.486 (2)	C5—C6	1.510 (3)
N2—C4	1.487 (2)	C5—H5A	0.9900
N2—H2	0.9300	C5—H5B	0.9900
N3—C6	1.485 (2)	C6—H6A	0.9900
N3—H3A	0.9200	C6—H6B	0.9900
N3—H3B	0.9200	N11—C11	1.162 (2)
C1—C2	1.512 (2)	C11—S11	1.6362 (16)
C1—H1C	0.9900	N21—C21	1.161 (2)
C1—H1D	0.9900	C21—S21	1.6280 (17)
			
N21—Co1—N3	107.58 (6)	C1—C2—C3	114.74 (14)
N21—Co1—N1	109.34 (6)	C1—C2—H2A	108.6
N3—Co1—N1	142.97 (5)	C3—C2—H2A	108.6
N21—Co1—N11	99.33 (6)	C1—C2—H2B	108.6
N3—Co1—N11	89.99 (6)	C3—C2—H2B	108.6
N1—Co1—N11	86.80 (5)	H2A—C2—H2B	107.6
N21—Co1—N2	90.27 (6)	N2—C3—C2	113.84 (13)
N3—Co1—N2	88.24 (6)	N2—C3—H3C	108.8
N1—Co1—N2	88.88 (5)	C2—C3—H3C	108.8
N11—Co1—N2	170.33 (6)	N2—C3—H3D	108.8
C1—N1—Co1	116.24 (10)	C2—C3—H3D	108.8
C1—N1—H1A	108.2	H3C—C3—H3D	107.7
Co1—N1—H1A	108.2	N2—C4—C5	114.03 (15)
C1—N1—H1B	108.2	N2—C4—H4A	108.7
Co1—N1—H1B	108.2	C5—C4—H4A	108.7
H1A—N1—H1B	107.4	N2—C4—H4B	108.7
C3—N2—C4	109.42 (13)	C5—C4—H4B	108.7
C3—N2—Co1	113.85 (10)	H4A—C4—H4B	107.6
C4—N2—Co1	113.08 (10)	C6—C5—C4	115.58 (16)
C3—N2—H2	106.7	C6—C5—H5A	108.4
C4—N2—H2	106.7	C4—C5—H5A	108.4
Co1—N2—H2	106.7	C6—C5—H5B	108.4
C6—N3—Co1	115.82 (11)	C4—C5—H5B	108.4
C6—N3—H3A	108.3	H5A—C5—H5B	107.4
Co1—N3—H3A	108.3	N3—C6—C5	111.42 (15)
C6—N3—H3B	108.3	N3—C6—H6A	109.3
Co1—N3—H3B	108.3	C5—C6—H6A	109.3
H3A—N3—H3B	107.4	N3—C6—H6B	109.3
N1—C1—C2	110.50 (13)	C5—C6—H6B	109.3
N1—C1—H1C	109.5	H6A—C6—H6B	108.0
C2—C1—H1C	109.6	C11—N11—Co1	167.72 (14)
N1—C1—H1D	109.5	N11—C11—S11	179.86 (18)
C2—C1—H1D	109.5	C21—N21—Co1	174.01 (14)
H1C—C1—H1D	108.1	N21—C21—S21	177.91 (15)

### Hydrogen-bond geometry (Å, °)

**Table:**

*D*—H···*A*	*D*—H	H···*A*	*D*···*A*	*D*—H···*A*
N1—H1A···S21^i^	0.92	2.82	3.6177 (14)	145
N1—H1B···S11^ii^	0.92	2.80	3.5665 (14)	142
N2—H2···S11^iii^	0.93	2.69	3.5903 (15)	162
N3—H3A···S21^iv^	0.92	2.69	3.5839 (16)	165

Symmetry codes: (i) −*x*+3/2, *y*−1/2, −*z*+3/2; (ii) −*x*+2, −*y*+1, −*z*+1; (iii) −*x*+1, −*y*+1, −*z*+1; (iv) *x*−1/2, −*y*+3/2, *z*−1/2.
